# A Cross-Cultural Examination of Intrasexual Competition: The Links Between Mating Competition, Aggression, Appearance Enhancement, and Sexuality

**DOI:** 10.1007/s10508-026-03464-8

**Published:** 2026-06-22

**Authors:** Norbert Meskó, Adam C. Davis, Steven Arnocky, Fanni Őry, Katalin Orsolya Burghardt, Dóra Eszter Kiss, Kevin Efrain Tololiu, Juneman Abraham, Hyemin Han

**Affiliations:** 1 Faculty of Humanities and Social Sciences, Institute of Psychology, University of Pécs, Pécs, 7624 Hungary; 2 Department of Social Sciences, Canadore College, North Bay, ON Canada; 3 Department of Psychology, Nipissing University, North Bay, ON Canada; 4 Department of Psychology, Faculty of Humanities, Bina Nusantara University, Jakarta, Indonesia; 5 Educational Psychology Program, University of Alabama, Tuscaloosa, AL USA

**Keywords:** Intrasexual mating competition, Cross-cultural analysis, Sensation seeking, Aggressiveness, Beauty enhancement, Sexuality

## Abstract

In the context of mating, individuals of the same sex often act as rivals in the pursuit, attraction, and retention of desirable partners. This study explored the relationships between intrasexual competition and various aspects of human mating psychology across three countries: Canada, Hungary, and Indonesia. A total of 661 adults (including women, men, non-binary, and gender-unspecified individuals) completed an online questionnaire assessing sensation seeking, aggression, beauty-enhancing behavior, openness to cosmetic surgery, sexual motivation, and sociosexuality. Hypotheses were tested via Bayesian multilevel modeling. Measurement invariance testing and alignment procedures were conducted to address potential cross-cultural non-invariance. Results indicated that the superiority enjoyment component of intrasexual competition showed consistent positive associations with the examined psychological variables. Associations involving inferiority frustration were generally weaker and less consistent. The findings for openness to cosmetic surgery, sociosexuality, and aggression replicate prior research, whereas the links with sensation seeking, beauty-enhancing behavior, and sexual motivation extend the literature. Cross-national comparisons revealed no significant country differences in superiority enjoyment, whereas Canadian participants scored significantly lower than Hungarian and Indonesian participants in inferiority frustration, with no significant difference between the latter two groups. Overall, the findings suggest that intrasexual competition—particularly its superiority enjoyment component—shows consistent associations with mating-relevant psychological traits across cultural contexts, even when mean levels differ between societies.

**Supplementary Information:**

The online version contains supplementary material available at 10.1007/s10508-026-03464-8.

## Introduction

### Intrasexual Competition

From an evolutionary standpoint, intrasexual competition refers to competition with same-sex others for access to and retention of mates (Andersson, 1994; Buss, 1988; Krems et al. 2023; Varella et al. 2017). During mate selection, individuals must calibrate their behavior in response to both potential partners and same-sex rivals, whose presence shapes perceived mating opportunities and constraints (Davis & Arnocky, 2022; Krems et al. 2023; Valentova et al. 2022; Walter et al. 2021). Two primary strategies have been described in the literature: self-promotion and competitor derogation (Bendixen & Kennair, 2015; Bleske-Rechek & Buss, 2006; Buss, 1988; Buss & Dedden, 1990; Karimi-Malekabadi et al. 2019; Schmitt, 2002; Schmitt & Buss, 1996; Vaillancourt, 2013). Self-promotion involves enhancing one’s own mate value, whereas competitor derogation aims to reduce the perceived value of rivals. These strategies may operate both before and after mate selection and are not restricted to one sex.

Evolutionary theories suggest that individuals who engage more intensely in intrasexual competition may exhibit behaviors and tendencies aimed at maximizing their reproductive success, including sensation seeking, aggression, beauty enhancement, openness to cosmetic surgery, unrestricted sociosexuality, and heightened sexual motivation. The next section introduces the psychological constructs that were analyzed in this study and their association with intrasexual competition. The final section focuses on the particularities of intrasexual competition across cultures.

### Sensation Seeking

Sensation seeking is a personality trait characterized by the pursuit of novel and intense experiences and is shaped by genetic, biological, and social factors (Zuckerman et al. 1980). Individuals high in sensation seeking tend to engage more frequently in risk-taking and exploratory behaviors, including in sexual contexts (Henderson et al. 2005; Wiederman & Hurd, 1999; Zuckerman et al. 1976). In mating-related domains, such tendencies may translate into greater willingness to pursue opportunities, tolerate uncertainty, and engage in competitive environments, which conceptually links sensation seeking to intrasexual competition.

Positive associations have been found between sensation seeking and risky sexual behavior (Henderson et al. 2005), preferences for novel sexual experiences (Zuckerman et al. 1976), and extradyadic sexual behavior (Wiederman & Hurd, 1999). For instance, Seto and colleagues (1995) found that men’s sensation seeking positively correlated with total number of sexual partners, average number of partners in sexually active years, shorter time from meeting to sex, proportion of short-term past relationships, number of desired partners in the next year, and diversity of sexual activities participants had experienced or wished to experience. They also found a negative link with age at first intercourse, indicating a short-term mating strategy.

### Aggression

Aggression can function as a strategy to outcompete rivals by intimidating them or limiting their access to resources and potential mates (Archer, 2009; Buss & Dedden, 1990). Both direct and indirect forms of aggression have been observed in mating-relevant competitive contexts. Cross-cultural evidence suggests that individuals may direct aggression toward same-sex rivals when reproductive or relational resources are perceived as scarce (Burbank, 1987; Campbell, 2013). Thus, aggression may represent one behavioral expression of heightened intrasexual competitiveness.

### Beauty Enhancement

Investment in appearance-enhancing practices can function as a competitive strategy when physical attractiveness is relevant for mate value and social comparison (Davis & Arnocky, 2022; Mafra et al. 2020; Varella et al. 2017, 2022). Because physical appearance often serves as a salient cue in mating contexts, individuals higher in intrasexual competitiveness may be more motivated to enhance or signal desirable traits. Evolutionary accounts suggest that physical cues related to health, vitality, and reproductive potential have historically influenced mate preferences, which may render appearance-based strategies particularly salient in competitive environments. Although the specific forms of appearance investment may vary, beautification behaviors have been documented across sexes as strategies to augment perceived attractiveness and competitive standing (Blake et al. 2020; Kowal et al. 2022; Wang et al. 2021).

### Cosmetic Procedures

The willingness to undergo cosmetic surgery is a more extreme manifestation of beauty enhancement within the context of intrasexual competition (Arnocky & Piché, 2014). Individuals who perceive themselves to be in a highly competitive environment are more likely to pursue such interventions, which do appear to provide a reproductive advantage (Swami et al. 2009). For instance, women undergoing microfat transfer surgery effectively reduce their waist-to-hip ratio, leading to a perceived increase in physical attractiveness (Singh & Randall, 2007). Similarly, rhinoplasty has been shown to enhance physical attractiveness, contributing to more favorable evaluations of an individual's personality (Cash & Horton, 1983). Women who undergo breast augmentation surgery also report being more attractive to those around them than before the procedure (Murphy et al. 2009).

Individuals higher in intrasexual competitiveness have been found to exhibit more positive attitudes toward cosmetic surgery (Thornton et al. 2013). Garza and Pazhoohi (2023) found that women with higher levels of intrasexual competition were more likely to enhance their physical appearance when exposed to images of potential competitors with attractive features.

Cosmetic surgeries may enhance cues to youthfulness, symmetry, or vitality, which can increase perceived attractiveness and influence mate selection and competitive dynamics (Ajitha & Sivakumar, 2017; Bonell et al. 2023; Frederick et al. 2016; Garza & Pazhoohi, 2023). Recent studies supporting this connection indicate that even contemplating cosmetic surgery regulates women’s mate preferences and mate retention (Ajitha & Sivakumar, 2017; Arnocky & Piché, 2014; Bradshaw et al. 2019). Bradshaw et al. (2019) found that women with heightened short-term mating effort were more accepting of costly appearance-enhancing techniques (e.g., cosmetic surgery) but not lower-cost appearance enhancements (e.g., facial cosmetics). Furthermore, both male and female participants inferred that an individual’s cosmetic surgical interventions served as a means for increasing short-term mating effort.

### Sexual Motivation

Sexual motivation refers to the range of psychological reasons that lead individuals to engage in sexual behavior, including personal, relational, and regulatory motives (Meskó et al. 2022a, 2022b, 2022c; Meston & Buss, 2007). Within competitive mating contexts, sexual behavior may serve multiple functions, such as pursuing personal gratification, strengthening relationships, or coping with emotional states. Therefore, variation in sexual motivation may be conceptually linked to differences in intrasexual competitiveness.

Limited research has explored the connection between sexual motivation and intrasexual competition, and these studies have mainly revealed indirect findings, especially among men. Bleske-Rechek and Buss (2006) found that men’s behavior related to mating efforts (e.g., frequent propositions and sex) correlated with how they perceived the effectiveness of masculine behavior, belittling a rival’s dominance and attractiveness, and challenging the dominance of competitors. In other words, men with higher mating effort employed relatively more intrasexual competition tactics. Mating effort can be directed toward short-term mating, including a greater intention to engage in sexual relationships with different partners, as well as long-term mating, such as a greater use of mate retention tactics (Gangestad & Simpson, 2000). In a study by Massar and Buunk (2009), male participants subliminally primed with sexual or commitment-related words were subsequently exposed to a romantic rival. They found that sexual desire modulated men’s propensity for intrasexual competition: Those with higher sexual desire felt more threatened by rivals in the sexual context, whereas men with lower sexual desire felt more threatened in the commitment context. Despite limited direct evidence, it is plausible that individuals more engaged in mating-related competition may report stronger or more diverse motivations for engaging in sexual behavior (Birnbaum et al. 2014).

### Sociosexuality

The combination of traits promoting short-term mating, including an unrestricted sociosexual orientation (e.g., a greater desire to have sex outside of committed relationships), encourages the use of riskier and antagonistic intrasexual competitive tactics. For instance, Simpson et al. (1999) had participants compete with a same-gender individual for a lunch date with an attractive opposite-gender other. They found that men with a short-term mating orientation (unrestricted sociosexuality) reported more direct intrasexual competition tactics (e.g., asserting superiority) than men with a long-term mating orientation (restricted sociosexuality) who resorted more to self-promotion strategies (e.g., using humor). Davis et al. (2023b) found that unrestricted sociosexuality, particularly the desire aspect, positively predicted same-sex indirect aggression, and that intrasexual competitiveness positively mediated this link in Canadian adults. Thus, individuals with a short-term mating orientation, especially those with unrestricted desires, engage in more indirect aggression toward same-sex peers, and this relation is partly explained by their tendency to be competitive with same-sex rivals for social and mating resources.

Under certain circumstances, intrasexual competition and unrestricted sociosexuality might enhance individual reproductive success, but these riskier strategies can contribute to poorer well-being. In a Brazilian study (Mafra et al. 2021) of heterosexual women, it was found that higher well-being negatively predicted intrasexual competition and sociosexuality. Unrestricted sociosexuality and intrasexual competition have also been positively linked to distress over not participating in salient social events and activities, referred to as fear of missing out (FoMO), in American adults (Davis et al. 2023a). Taken together, these results suggest that unrestricted sociosexual orientation may be related to heightened intrasexual competition.

### Cultural Differences in Intrasexual Competitiveness

Intrasexual competition has been examined across a variety of national contexts, including samples from Argentina, Brazil, Canada, Chile, Switzerland, the Netherlands, and Uruguay (e.g., Buunk, 2022; Buunk et al. 2010; Fiacco et al. 2019; Mafra et al. 2021; Massar & Buunk, 2009). However, relatively few studies have explicitly aimed at direct cross-national comparisons using the same measurement framework.

Buunk and Fisher (2009), who developed the Intrasexual Competition Scale (ICS), demonstrated that the scale showed acceptable reliability and cross-national equivalence in Dutch and Canadian university samples, although associations with sociosexuality differed between countries. Subsequent research has linked intrasexual competition to personality traits and mating-related variables in other cultural contexts (Buunk et al. 2017), suggesting both cross-cultural robustness and contextual variability in its correlates.

Beyond individual-level traits, broader cultural dimensions may also shape competitive attitudes toward same-sex peers. For instance, vertical individualism and lower gender equality have been associated with higher intrasexual competitiveness (Buunk, 2022). Moreover, recent large-scale cross-national research has proposed multidimensional models of intrasexual competition and reported cultural variation in specific components of the construct (Jonason et al. 2023).

Taken together, existing findings suggest that although intrasexual competition appears measurable across diverse cultural contexts, both its mean levels and its associations with related psychological constructs may vary. These mixed findings highlight the need for further comparative research employing consistent measurement approaches across countries.

### Present Study

Research indicates that the degree of intrasexual competition may vary across countries alongside different cultural dimensions. However, there is limited cross-cultural work on intrasexual competition, and it is unclear whether the links between intrasexual competitiveness with various facets of human mating, such as sociosexuality and aggression, vary between countries. Moreover, the general relations between intrasexual competition with sensation seeking, appearance enhancement, and sexual motives are unclear. The current study involves three countries (Canada, Hungary, and Indonesia) that are geographically and culturally distinct. Canada, located in North America, embraces an individualistic value system and aligns more with WEIRD cultures. Indonesia, situated in Southeast Asia, predominantly holds a collectivistic value system and is categorized among non-WEIRD cultures. Hungary, located in Central Europe, falls between the other two in terms of geography but culturally might be closer to Canada due to its individualistic values.

## Aims of the Study, Hypotheses

### Hypothesis 1 |

 Intrasexual competition will correlate positively with sensation seeking.

### Hypothesis 2

| Intrasexual competition will correlate positively with aggression.

### Hypothesis 3

| Intrasexual competition will correlate positively with all facets of acceptance of cosmetic surgery.

### Hypothesis 4

| Intrasexual competition will correlate positively with beauty-enhancing behavior.

### Hypothesis 5.1

| Personal goal attainment sexual motives will correlate positively with intrasexual competition.

### Hypothesis 5.2

| Relational reasons sexual motives will correlate positively with intrasexual competition.

### Hypothesis 5.3

| Sex as coping sexual motives will correlate positively with intrasexual competition.

### Hypothesis 6

| Intrasexual competition will positively correlate with unrestricted sociosexual desires, attitudes, and behavior.

### Hypothesis 7

| It is expected that intrasexual competition will be more pronounced in the collectivistic/tight countries than in the individualistic/loose countries. Thus, intrasexual competition will be the highest in Canada, followed by Hungary, then Indonesia.

## Method

### Participants and Procedure

Overall, 661 participants completed the online survey from the three participating countries: 124 individuals from Canada, 272 from Hungary, and 96 from Indonesia. To increase the number and diversity of respondents, the survey link was also sent to students attending the international BA Psychology program at the University of Pécs, which added another 169 respondents from different countries, such as Australia (1), Bangladesh (2), and India (4), but most did not specify their country (151).

The final sample included 497 (75.2%) women, 132 (20.0%) men, 29 (4.4%) non-binary individuals, and 3 (.5%) who chose “prefer not to say” Regarding sex at birth, 523 (79.1%) were women, 137 (20.7%) were men, and 1 (.2%) individual was intersex. The participants were aged 18 to 67 years (*M* = 27.3, *SD* = 10.05, *Mdn* = 23). Regarding intimate partner relationship, 240 (36.3%) participants were single (not dating anyone), 53 (8.0%) were single (not in a long-term relationship, occasional sexual encounters), 144 (21.8%) were in a committed relationship but not living together, 97 (14.7%) were living together with a partner but not in marriage or official cohabited relationship, 108 (16.3%) were living with a partner in marriage or official cohabited relationship, and 19 (2.9%) answered “other” and named different partnered situations (e.g., “polyamorous,” “widow”).

Hungarian participants completed the survey in their own language, whereas everyone else completed the survey in English. All the scales used in the survey had a previously published Hungarian version except for the ICS. A preliminary step was to translate the questions and instructions of the ICS into Hungarian by the authors. The accuracy of the Hungarian version was confirmed using the standard back-translation technique (Brislin, 1980; Hambleton & De Jong, 2003; Muñiz et al. 2013), where an independent translator, not associated with the study, translated the questions and instructions back into English. Any discrepancies that arose during the back-translation were resolved through collaboration between the two translators. Online data were collected between May and November 2023 using the cloud-based surveying platform Qualtrics. Originally, the intention was to send the research link to a sample of university students; however, as the target of around 200 participants per country was not achieved, data collection was expanded to include social media platforms where participants were invited to share the link on their own social media networks.

The determination of sample size, data exclusions, manipulations, and all procedures in the study were reported in accordance with the Journal Article Reporting Standards (JARS; Kazak, 2018). All data, analysis code, and research materials are available at https://osf.io/7a6qr/?view_only=be4c9ef8d3744cfd951ded2205025f88. Data were analyzed using *R*, version 4.0.0 (R Core Team, 2020). Although the study was guided by theory-driven hypotheses, these hypotheses and analytic decisions were not preregistered.

### Measures

#### Intrasexual Competition Scale (ICS)

The ICS (Buunk & Fisher, 2009) is a 12-item self-report instrument designed for measuring individual differences in intrasexual competitiveness. A recent analysis [85] explored two subscales, namely superiority enjoyment and inferiority frustration, that identify distinct dimensions of intrasexual competition. As one anonymous reviewer suggested we consider a one-factor model instead of the two-factor model, we performed CFA to examine which model is superior to the other. The CFA result confirmed that the two-factor model, RMSEA = .053, SRMR = .068, CFI = .976, was better than the one-factor model, RMSEA = .087, SRMR = .091, CFI = .934. The subsequent χ^2^ test also supported that χ^2^ (4) = 176.15, p < .001. Given the result, we decided to continue to use the two-factor. The items of the superiority enjoyment subscale reflect the pursuit of social superiority over same-sex rivals (e.g., “I want to be just a little better than other men/women”). Items in the inferiority frustration subscale capture a sense of frustration arising from encounters with same-sex rivals whom the person perceives as a more desirable mate (e.g., “I wouldn’t hire a very attractive man/woman as a colleague” Or “I just don’t like very ambitious men/women”). Each item was rated on a seven-point scale ranging from “Not typical at all” (1) to “Very typical” (7). Higher scores, computed by averaging the items, indicated higher levels of intrasexual competition on both subscales.

#### Brief Sensation Seeking Scale (BSSS-8)

The BSSS-8 (Hoyle et al. 2002; Hungarian version: Mayer et al. 2012) is an 8-item self-report instrument designed to measure individual differences in sensation seeking. The scale includes four subscales: Experience Seeking, Boredom Susceptibility, Thrill and Adventure Seeking, and Disinhibition. In our current research, we used this instrument as a single composite scale (e.g., “I would love to have new and exciting experiences, even if they are illegal”). Each item was rated on a five-point scale ranging from “Strongly disagree” (1) to “Strongly agree” (5). Items were averaged to calculate a BSSS-8 total score, with higher scores describing greater levels of sensation seeking.

#### Buss–Perry Aggression Questionnaire (BPAQ)

The BPAQ (Bryant & Smith, 2001; Hungarian version: Gerevich et al. 2007) is a 12-item self-report instrument which includes four subscales for measuring individual differences in aggressiveness. The inventory provides sum scores for subscales: physical aggression (e.g., “Given enough provocation, I may hit another person”), verbal aggression (e.g., “I can’t help getting into arguments when people disagree with me”), anger (e.g., “I have trouble controlling my temper”), and hostility (e.g., “At times I feel I have gotten a raw deal out of life”). Each item was rated on a four-point scale ranging from “Strongly disagree” (1) to “Strongly agree” (4). Higher summative scores denote higher levels of aggression.

#### Acceptance of Cosmetic Surgery Scale (ACSS)

The ACSS (Henderson-King & Henderson-King, 2005; Hungarian version: Meskó & Láng, 2021) is a 15-item self-report instrument designed to measure individual differences in openness to cosmetic procedures. The scale includes three subscales: intrapersonal (e.g., “It makes sense to have minor cosmetic surgery rather than spending years feeling bad about the way you look”), social motivations (e.g., “I would seriously consider having cosmetic surgery if my partner thought it was a good idea”), and consider (e.g., “In the future, I could end up having some kind of cosmetic surgery”). Each item was rated on a seven-point scale ranging from “Strongly disagree” (1) to “Strongly agree” (7). Items were averaged and higher total scores on the scale indicate higher levels of openness to cosmetic surgery.

#### Beauty Enhancement Behavior Scale (BEBS)

The BEBS (Kowal et al. 2022) is an 8-item self-report instrument designed to measure individual differences in non-cultural behaviors associated with enhancing beauty. The BEBS includes eight common categories of beauty-enhancing behaviors: (1) applying makeup, (2) body hygiene, (3) using cosmetics, (4) exercising, (5) hair grooming, (6) clothing style, (7) following a specific diet, and (8) other (where participants could describe what other activities they perform). Participants were asked to specify both the occurrence and frequency of their engagement in a designated beauty-enhancing activity. Clear instructions were provided, emphasizing that participants should select the specified activity solely if their motivation was to enhance their appearance (excluding other reasons like health considerations). In each category, participants were instructed to quantify the time on a scale dedicated to performing the designated activity on a typical day. The BEBS has been developed in 43 languages, including English and Hungarian. Higher scores denote more time spent on improving personal attractiveness and greater beauty-enhancing behavior.

#### 15-item version of the Reasons for Having Sex Questionnaire (YSEX?-15)

The YSEX?-15 (Meskó et al. 2022b) is a 15-item Hungarian self-report measure of sexual motivation derived from the original American YSEX? questionnaire developed by Meston and Buss (2007). The questionnaire includes three subscales: personal goal attainment (e.g., “I wanted to seek experience”), relational reasons (e.g., “I wanted to comfort the other person”), and sex as coping (e.g., “I wanted to recharge myself”). Participants were requested to indicate how frequently each of the listed reasons led them to have sexual intercourse in the past. If someone had not yet had sex, they were asked to indicate the likelihood that each of the listed reasons would lead them to have sex. Each item was rated on a 5-point scale ranging from “None of my sexual experiences” (1) to “All of my sexual experiences” (5). Items were averaged with higher scores indicating higher sexual motivation.

#### Revised Sociosexual Orientation Inventory (SOI-R)

The SOI-R (Penke & Asendorpf, 2008; Hungarian version: Meskó et al. 2014) is a 9-item self-report instrument including three subscales for measuring individual differences in sociosexuality (uncommitted sexuality): behavior (e.g., “With how many different partners have you had sexual intercourse without having an interest in a long-term committed relationship with this person?”); attitudes (e.g., “I can imagine myself being comfortable and enjoying «casual» sex with different partners”); and desire (e.g., “In everyday life, how often do you have spontaneous fantasies about having sex with someone you have just met?”). The participants rated each item on 9-point scales: “1 = 0 to 9 = 20 or more” for sociosexual attitudes; and “1 = Strongly agree to 9 = Once a day” for sociosexual desire. Higher overall scores indicated a more unrestricted sociosexual orientation.

### Analysis Plans

#### Measurement Invariance Test and Measurement Alignment

Because we intended to conduct cross-national examinations with data collected with survey forms translated into different languages, we assessed measurement invariance before testing our hypotheses (Putnick & Bornstein, 2016). A measurement invariance test is required to ensure that different versions of the same scale, different languages in the present study, measure the intended latent factor invariantly (Fischer & Karl, 2019). We examined whether all the employed scales were invariant across the English and Hungarian versions. In addition to these tests for cross-group validity check, we examined the internal consistency of each measure via Cronbach α, which indicated acceptable reliability.

Measurement invariance can be examined via multigroup confirmatory factor analysis (MG-CFA). We employed an R package, *lavaan*, for this test (Rosseel, 2012). There are four different levels of invariance (configural, metric, scalar, and residual) in terms of the extent to which contrasts across groups are applied while testing a measurement model of interest (Putnick & Bornstein, 2016). Configural invariance, the most lenient invariance, only assumes that the measurement model is identical across different groups. Metric invariance additionally requires the equal factor loadings. For scalar invariance, the equal intercept requirement should be satisfied. Residual invariance, the strictest invariance, is established when the equal residuals are supported by data. To compare group means and conduct regression analysis or other types of inferences involving multiple groups, at the least, scalar invariance should be established as a necessary condition (Han, 2024a).

Whether each level of invariance was supported by data could be tested by examining model fit indicators, RMSEA, SRMR, and CFI. First, we examined whether configural invariance was supported by data by examining the model fit indicators after conducting MG-CFA. Following the general guidelines, we employed RMSEA and SRMR < .80 and CFI ≥ .90 as criteria to determine whether those were deemed acceptable (Han, 2024a; Rachev et al. 2021). Metric, scalar, and residual invariances were tested by examining the extent to which the model fit indicators changed when additional constraints were added to the model while conducting MG-CFA. We concluded that metric invariance was supported when ΔRMSEA <  + .015, ΔSRMR <  + .030, and ΔCFI ≥ -.010. For scalar and residual invariances, we employed ΔRMSEA <  + .015, ΔSRMR <  + .015, and ΔCFI ≥ -.010 as criteria. Because the responses to the items were anchored to an ordinal scale, we used WLSMV estimator to minimize any potential bias during MG-CFA (Li, 2016).

If scalar invariance was not supported, we performed measurement alignment to address the non-invariance issue (Asparouhov & Muthén, 2014; Fischer & Karl, 2019). Measurement alignment is a method to adjust parameters, such as factor loadings and intercepts, to enable between-group comparisons for instances of non-invariance. The alignment routine automatically adjusts model parameters so that the degree of existing non-invariance is minimized. The automated process continuously seeks adjusted parameters until the optimal invariance pattern is achieved. The alignment procedure was conducted for each individual subscale of interest as the alignment technique is only capable of addressing one factor, not multiple factors, in a measurement model at a time (Fischer & Karl, 2019).

We employed the measurement alignment method with R following Han (2023, 2024a), and we adjusted factor loadings and intercepts via the alignment routine implemented in an R library, *sirt* (Robitzsch, 2023). The alignment process was performed for each scale. Once the procedure was completed, we examined whether the alignment method was able to address the existing non-invariant issue successfully by checking several indicators. First, we investigated the resultant *R*^*2*^ values, *R*^*2*^_*loadings*_, and *R*^*2*^_*intercepts*_ indicating the extent to which the non-invariance was absorbed via alignment in the cases of factor loadings and intercepts, respectively. When both exceeded 75%, we deemed that non-invariance in loadings and intercepts was successfully absorbed, so scalar invariance for comparison and inference was achieved (Asparouhov & Muthén, 2014). Second, we analyzed whether 25% or fewer items showed significant non-invariance after alignment for both factor loadings and intercepts (Fischer & Karl, 2019).

For hypothesis tests, latent factor scores were used (DiStefano et al. 2019). In the cases of scales that successfully achieved scalar invariance during MG-CFA without alignment, latent factor scores were calculated with *lavPredict* function implemented in *lavaan*, which estimates latent factor scores with factor loadings and intercepts resulting from CFA. If measurement alignment was conducted, we calculated latent factor scores using the adjusted factor loadings and intercepts by language group (Han, 2024a). We used an *R* function following Han (2024a) to calculate the scores.

One exception was the BEBS, which measured how long each participant spent on different types of beauty-enhancing behaviors, concrete behavioral outcomes, instead of a latent psychological construct. Hence, we used the composite score instead of the latent factor score after conducting MG-CFA or measurement alignment.

#### Hypothesis Testing

*Association testing* For general association tests, H1-6, we conducted Bayesian model exploration to examine which model was the best among possible candidate models (Blackburn et al. 2023). We employed a Bayesian approach in the present study because it enables direct testing of a hypothesis of interest instead of a null hypothesis and direct model comparison and exploration, which could not be done feasibly through conventional frequentist approach (Han, 2021). Furthermore, inflated false positives are not considered to be significant issues in Bayesian analysis even when correction methods are not applied, because it is based on statistical assumptions that are different from those for conventional frequentist analysis (Kruschke, 2010). Because we intended to test multiple hypotheses, this aspect of Bayesian analysis provided a unique benefit as we were concerned about the potential false positives.

Given data were collected across different countries, we examined whether the inclusion of random effects, random intercepts, and slopes improved the model (Blackburn et al. 2023; Han, 2023b). We employed multilevel modeling examining both fixed effects at the population level and random effects at the group level (Han, 2024b). Multilevel modeling including fixed and random effects is the most preferred way for hypothesis testing with multiple groups (Brown, 2021).

These hypothetical models were based on different assumptions. In the case of the most complex model, M3, the random-slope model, we assumed that the relationship between intrasexual competition and the dependent variables of interest could vary across cultural contexts, such that the strength of the association might be stronger in some countries and weaker in others.

To evaluate whether this level of complexity was warranted, we compared the random-slope model (M3) with a series of simpler alternatives, including a null model without predictors (M0), a simple model including intrasexual competition as a predictor (M1), and a random-intercept model allowing baseline differences across countries while constraining the association to be equal across contexts (M2). This stepwise comparison allowed us to assess whether cross-national variation in the associations provided additional explanatory value beyond differences in average levels alone.

Using multilevel modeling, for instance, in the case of H1, we compared these models:

M0 (null model): BSSS ~ 0 (intercept only).

M1 (simple model): BSSS ~ ICS.superiority.

M2 (random-intercept model): BSSS ~ ICS.superiority + (1|country).

M3 (random-slope model): BSSS ~ ICS.superiority + (1 + ICS.superiority |country).

Model comparison was conducted by calculating and comparing model Bayes factors (BFs; Wagenmakers et al. 2018). A BF indicates the extent to which one model is better compared to another model. For instance, BF_10_ indicates the degree to which M1 is better compared with M0. We calculated all model BFs, BF_10_, BF_20_, and BF_30_ for all hypotheses via Bayesian multilevel modeling implemented in brms package with the default prior distribution for model exploration, Cauchy (0, 1) (Bürkner, 2017; Rouder & Morey, 2012). For decision making, we assumed that one specific model was significantly better than others when the responsive 2log(modelBF) ≥ 2. 2log(BF) ≥ 2 has been used as a criterion to determine whether evidence supported one hypothetical model better than the other positively (Kass & Raftery, 1995). If the model BF comparing two specific models did not exceed the threshold, then we calculated Bayesian *R*^*2*^ values to determine which model to be further examined (Bürkner, 2017). To ensure successful model convergence, we only included data collected from countries where 30 or more participants completed the survey (Blackburn et al. 2022). All variables were standardized to obtain standardized coefficients as indicators for effect sizes.

Once the best model was identified, we examined the BF of the predictor of interest; for instance, ICS superiority in the case of H1 was higher than the threshold, 2log(BF) ≥ 2. Similar to the case of model exploration, 2log(BF) ≥ 2 is used as a threshold to conclude whether evidence positively supports an alternative hypothesis versus a null hypothesis (Kass & Raftery, 1995). In this case, the alternative hypothesis of interest became whether the effect of the predictor was significantly higher (or lower) than zero.

*Cross-national comparison of intrasexual competition.* For testing H7, we compared the mean score between examined countries with the calculated latent factor scores (DiStefano et al. 2019). If scalar invariance was established, we compared the group mean scores estimated from the resultant MG-CFA model with *lavPredict* in *lavaan*. If scalar invariance was not supported, we used the group mean scores estimated via measurement alignment (Fischer & Karl, 2019; Han, 2024a). Like association tests, only data collected from countries where 30 or more participants completed the survey were examined to ensure convergence during model testing. We conducted *t*-tests to test group differences with the estimated group means and standard deviations. The resultant *p*-values were corrected for false discovery rates to minimize the possibility of Type I error.

## Results

### Measurement Invariance Test and Measurement Alignment

The results of internal consistency, measurement invariance, and measurement alignment tests are reported in Table 1. Both subscales for the ISC demonstrated good internal consistency. The superiority and inferiority subscales could not achieve scalar invariance. Thus, we performed measurement invariance. The measurement invariance successfully addressed the non-invariance found from MG-CFA.

**Table 1:** Invariance levels and criteria

Invariance level	Assumptions	Criteria
Configural	The identical measurement model across different groups	RMSEA and SRMR < .80 and CFI ≥ .90
Metric	Equal factor loadings	ΔRMSEA < + .015, ΔSRMR < + .030, and ΔCFI ≥ -.010
Scalar	Equal factor intercepts	ΔRMSEA < + .015, ΔSRMR < + .015, and ΔCFI ≥ -.010
Residual	Equal residuals	ΔRMSEA < + .015, ΔSRMR < + .015, and ΔCFI ≥ -.010

The BSSS demonstrated acceptable internal consistency. Since even configural invariance was not supported, we performed measurement to correct for non-invariance.

In the BPAQ, all subscales showed acceptable internal consistency except for the verbal aggression subscale. Because all subscales did not demonstrate scalar invariance, we conducted measurement alignment, which successfully addressed the non-invariance issue in all subscales.

The ACSS demonstrated good internal consistency in all subscales. However, all subscales failed to achieve even configural invariance, so measurement alignment was performed to address the non-invariance issue.

The resultant α values suggested the YSEX?-15H possessed good internal consistency in all subscales. The results from measurement invariance tests indicated that measurement alignment was required. Measurement alignment addressed the problem.

For all subscales of the SOI-R, the internal consistency was good. When MG-CFA was performed, only the sociosexual desire subscale achieved the strictest invariance, residual invariance, which was more than the required minimum (scalar invariance). Thus, we conducted measurement alignment for two other subscales: the sociosexual behavior and attitudes subscales. The alignment process addressed non-invariance for the two subscales.

### Hypothesis Testing

#### Association Between Intrasexual Competition and Sensation Seeking (H1)

When we examined the association between ICS superiority enjoyment and sensation seeking, as shown in Table 2, the best model was the random-intercept model (M2), indicating that mean levels of sensation seeking differed across countries, but the positive association between superiority enjoyment and sensation seeking was comparable across cultures. Intrasexual competition superiority was found to be positively associated with sensation seeking, β = .24, *SE* = .04, Bayesian 95% CI = [.17, .31], 2log(BF) = Infinite (see Table 3). No significant association emerged between inferiority frustration and sensation seeking.

**Table 2:** Results from measurement invariance test and measurement alignment

		MG-CFA						Measurement alignment			
	α	RMSEA	SRMR	CFI	ΔRMSEA	ΔSRMR	ΔCFI	*R*^*2*^_*loadings*_	*R*^*2*^_*intercepts*_	% of non-invariance loadings	% of non-invariance intercepts
Intrasexual Competition Scale											
Superiority enjoyment	.81							99.72%	99.86%	0.00%	0.00%
Configural invariance		.041	.017	.997							
Metric invariance		.033	.026	.996	−.009	.009	.000				
Scalar invariance		.092	.049	.957	.060	.023	−.039				
Inferiority frustration	.82							99.47%	99.59%	0.00%	0.00%
Configural invariance		.089	.073	.856							
Brief Sensation Seeking Scale	.79							99.39%	99.46%	0.00%	0.00%
Configural invariance		.099	.058	.883							
Buss–Perry Aggression Questionnaire											
Physical aggression	.68							98.28%	99.58%	0.00%	0.00%
Configural invariance		.000	.000	1.000							
Metric invariance		.083	.037	.955	.083	.037	−.045				
Verbal aggression	.57							96.29%	97.57%	0.00%	0.00%
Configural invariance		.000	.000	1.000							
Metric invariance		.150	.042	.924	.150	.042	−.076				
Anger	.64							98.70%	99.33%	0.00%	0.00%
Configural invariance		.000	.000	1.000							
Metric invariance		.074	.026	.985	.074	.026	−.015				
Hostility	.61							99.62%	99.41%	0.00%	0.00%
Configural invariance		.000	.000	1.000							
Metric invariance		.000	.013	1.000	.000	.013	.000				
Scalar invariance		.181	.075	.750	.181	.062	−.250				
Acceptance of Cosmetic Surgery Scale											
Interpersonal	.91							99.93%	99.84%	0.00%	0.00%
Configural invariance		.092	.019	.982							
Social	.88							99.30%	99.83%	0.00%	0.00%
Configural invariance		.126	.045	.923							
Consider	.92							99.72%	99.97%	0.00%	0.00%
Configural invariance		.109	.021	.982							
Reasons for Having Sex Questionnaire											
Personal goal attainment	.70							98.26%	99.73%	0.00%	0.00%
Configural invariance		.107	.113	.902							
Relational reasons	.82							99.42%	99.49%	0.00%	0.00%
Configural invariance		.113	.042	.961							
Sex as coping	.74							98.21%	99.57%	0.00%	0.00%
Configural invariance		.050	.032	.972							
Metric invariance		.069	.055	.925	.019	.023	−.047				
Revised Sociosexual Orientation Inventory											
Sociosexual behavior	.83							98.87%	99.90%	0.00%	0.00%
Configural invariance		.000	.000	1.000							
Metric invariance		.163	.077	.900	.163	.077	−.100				
Sociosexual attitude	.81							98.37%	99.90%	0.00%	0.00%
Configural invariance		.000	.000	1.000							
Metric invariance		.137	.031	.980	.137	.031	−.020				
Sociosexual desire	.87							Not required	Not required	Not required	Not required
Configural invariance		.000	.000	1.000							
Metric invariance		.000	.006	1.000	.000	.006	.000				
** Scalar invariance**		**.000**	**.006**	**1.000**	**.000**	**.001**	**.000**				
** Residual invariance**		**.000**	**.029**	**1.000**	**.000**	**.023**	**.000**				

*Note*: When the minimum requirement for MG-CFA, scalar invariance, was achieved, the MG-CFA results were bolded

**Table 3:** Bayesian hypothesis-specific models predicting intrasexual competitiveness. Each row reflects the predictors included in the model corresponding to the stated hypothesis (H1–H6); values represent Bayesian parameter estimates rather than correlations

Hypothesis		Whole data		
		2log(BF_10_)	2log(BF_20_)	2log(BF_30_)
H1	BSSS Sensation Seeking	15.97	33.20	28.88
H2	BPAQ Physical Aggression	2.21	90.67	92.41
	BPAQ Verbal Aggression	40.74	63.22	61.78
	BPAQ Anger	16.66	23.50	18.85
	BPAQ Hostility	21.61	46.38	44.99
H3	ACSS Interpersonal	19.18	14.94	10.87
	ACSS Social	30.66	26.96	24.56
	ACSS Consider	11.98	13.46	9.37
H4	BEBS Beauty Enhancement	−5.94	47.32	43.85
H5	YSEX?-15 Goal Attainment—ICS Superiority	16.30	45.00	42.56
	YSEX?-15 Goal Attainment—ICS Inferiority	8.72	38.81	40.22
	YSEX?-15 Relational Reasons—ICS Superiority	4.91	52.35	53.97
	YSEX?-15 Relational Reasons—ICS Inferiority	.62	49.46	51.86
	YSEX?-15 Sex as Coping—ICS Superiority	3.47	.40	-1.64
	YSEX?-15 Sex as Coping—ICS Inferiority	8.53	6.57	5.13
H6	SOI-R Behavior—ICS Superiority	4.04	33.89	32.08
	SOI-R Behavior—ICS Inferiority	.86	32.19	27.34
	SOI-R Attitude—ICS Superiority	9.51	83.28	77.57
	SOI-R Attitude—ICS Inferiority	−2.56	71.89	65.47
	SOI-R Desire—ICS Superiority	21.42	51.34	44.92
	SOI-R Desire—ICS Inferiority	6.58	37.60	31.72

Note: ICS = Intrasexual Competition Scale; BSSS = Brief Sensation Seeking Scale; BPAQ = Buss–Perry Aggression Questionnaire; ACSS = Acceptance of Cosmetic Surgery Scale; BEBS = Beauty-Enhancing Behavior Scale; YSEX?-15 = Brief Version of Reasons for Having Sex Questionnaire; SOI-R = Revised Sociosexual Orientation Inventory

#### Association Between Intrasexual Competition and Aggression (H2)

We explored the best prediction model for each subscale of the BPAQ (see Table 2 for 2log[modelBF]). First, when we examined the association between superiority enjoyment and physical aggression, the best model was the random-slope model, suggesting that the strength of the association between superiority enjoyment and physical aggression varied across countries. However, 2log(BF_32_), the 2log of the difference between the BF of M3 (random-slope model) and M2 (random-intercept model), was not greater than 2, so we compared *R*^*2*^ values between M3 and M2, which were 23.12 and 23.13%, respectively. Thus, we examined the random-slope model. Thus, we retained the random-slope model, suggesting that the strength of the association between superiority enjoyment and physical aggression may vary across countries. The superiority enjoyment was found to be positively associated with physical aggression, β = .24, *SE* = .32, Bayesian 95% CI = [-.25, .70], 2log(BF) = 3.60.

Second, in the case of the verbal aggression, the best prediction model was the random-intercept model. So, we focused on the random-slope model, indicating that the association between superiority enjoyment and verbal aggression showed some cross-cultural variability. However, like the previous case, 2log(BF_32_) was not greater than 2, so we compared *R*^*2*^ values between M3 and M2, which were 17.40 and 16.18%, respectively. So, we focused on the random-slope model. ICS superiority was positively associated with verbal aggression, β = .31, *SE* = .33, Bayesian 95% CI = [-.21, .77], 2log(BF) = 4.24.

Third, when the Anger subscale was examined, the best model was the random-intercept model. ICS superiority was positively linked with anger, β = .24, *SE* = .05, Bayesian 95% CI = [.16, .31], 2log(BF) = Infinite.

Regarding the relationship between ICS superiority and hostility, the best model was the random-intercept model, indicating cross-cultural differences in mean hostility levels, but a consistent positive association with superiority enjoyment across countries. However, 2log(BF_21_) was not greater than 2, so we compared *R*^*2*^ values between M2 and M1, which were 14.37 and 13.07%, respectively. In this model, ICS was positively associated with hostility, β = .26, *SE* = .35, Bayesian 95% CI = [-.26, .76], 2log(BF) = 3.80.

Associations between inferiority frustration and the aggression subscales did not reach statistical significance.

#### Association Between Intrasexual Competition and Frequency of Beauty-enhancing Behaviors (H3)

When the BEBS was examined, the identified best model was the random-intercept model (see Table 2), indicating that mean levels of beauty-enhancing behavior differed across countries, while the positive association with superiority enjoyment was consistent. The random-intercept model supported a positive relation between superiority enjoyment and the frequency of beauty-enhancing behavior, whereas the association with inferiority frustration did not reach statistical significance, β = .06, *SE* = .04, Bayesian 95% CI = [-.01, .13], 2log(BF) = 4.57.

#### Association Between Intrasexual Competition and Openness to Cosmetic Surgery (H4)

We explored the best prediction models for three subscales of the ACSS (interpersonal, social, and consider) (see Table 2). First, when we examined the Interpersonal subscale, the fixed effect model was the best model, indicating that neither mean levels nor associations differed across countries. A positive association was supported between ICS superiority and the interpersonal subscale, β = .23, *SE* = .05, Bayesian 95% CI = [.16, .31], 2log(BF) = Infinite.

Second, when we examined the social subscale, the random-intercept model performed the best, indicating cross-cultural differences in mean levels of the Social subscale, but a consistent positive association with superiority enjoyment. In this model, the relation between the ICS superiority and ACSS Social subscale was positive, β = .28, *SE* = .04, Bayesian 95% CI = [.21, .35], 2log(BF) = Infinite.

Third, when the consider subscale was tested, 2log(BF_21_) did not exceed 2. Thus, we compared *R*^*2*^s between M2 (6.34%) and M1 (4.21%). *R*^*2*^s indicated that the random-intercept model (M2) was better, indicating variation in mean levels across countries, but similar associations with superiority enjoyment. In the random-intercept model, the association between ICS superiority and consider was positive, β = .20, *SE* = .05, Bayesian 95% CI = [.13, .28], 2log(BF) = Infinite.

Inferiority frustration was not significantly associated with any of the ACSS subscales.

#### Association Between Intrasexual Competition and Reasons for Having Sex (H5)

First, we examined the relationship between ICS and personal goal attainment. In the case of superiority enjoyment, the random-intercept model was the best model, indicating cross-cultural differences in mean levels, but similar associations with superiority enjoyment. The association was significantly positive, β = .21, *SE* = .04, Bayesian 95% CI = [.13, .28], 2log(BF) = Infinite. When the inferiority frustration subscale was tested, the random-slope model was best, indicating potential cross-cultural variation in the association between inferiority frustration and personal goal attainment. However, 2log(BF32) was not greater than 2, so we compared *R*^*2*^ values between M3 and M2, which were 14.11 and 11.70%, respectively. In the random-slope model, a positive association was found between inferiority and goal attainment, β = .09, *SE* = .06, Bayesian 95% CI = [.00, .19], 2log(BF) = 6.16.

Second, when the links between the ICS and YSEX-15H relational reasons subscale were examined, the random-slope model was found to be best, suggesting that the association between superiority enjoyment and relational reasons varied across countries. The difference between 2log(BF_30_) and 2log(BF_20_) did not exceed 2, so we examined *R*^*2*^s. In the case of superiority enjoyment, *R*^*2*^ of M3 and M2 were 16.30 and 14.43%, respectively. In M3 with the higher *R*^*2*^, the association was positive, β = .17, *SE* = .32, Bayesian 95% CI = [-.33, .66], 2log(BF) = 2.74. When the Inferiority subscale was tested, the random-slope model was also the best. In the model, a significant positive association between inferiority frustration and relational reasons was found, β = .12, *SE* = .33, Bayesian 95% CI = [-.38, .61], 2log(BF) = 2.05.

Third, when we examined the relationship between the ICS superiority subscale and sex as coping, the fixed effect model was found to be the best, indicating that both mean levels and associations were comparable across countries. In the model, superiority was positively related with sex as coping, β = .15, *SE* = .05, Bayesian 95% CI = [.07, .23], 2log(BF) = 14.39. Regarding inferiority, 2log(BF_21_) was not greater than 2, so we compared *R*^*2*^ values between M2 and M1, which were 4.73 and 3.44%, respectively. So, we focused on the random-intercept model, indicating cross-cultural differences in mean levels of sex as coping, but a consistent positive association with inferiority frustration, where inferiority frustration was also positively linked with sex as coping, β = .19, *SE* = .05, Bayesian 95% CI = [.11, .27], 2log(BF) = Infinite.

#### Association Between Intrasexual Competition and Sociosexuality (H6)

First, when we examined the associations between superiority enjoyment and sociosexual behavior, the prediction models were significantly better than the null model (see Table 2). However, the difference between 2log(BF_30_) and 2log(BF_20_) did not exceed 2, so we examined *R*^*2*^s. *R*^*2*^ of M3 and M2 was 11.93 and 10.81%, respectively. Thus, we decided to examine the random-slope model (M3), indicating that the strength of the association between superiority enjoyment and sociosexual behavior varied across countries. Superiority enjoyment was positively associated with sociosexual behavior, β = .15, *SE* = .26, Bayesian 95% CI = [-.24, .51], 2log(BF) = 3.14. In the case of the association with ICS inferiority, the best model was the random-intercept model, indicating cross-cultural differences in mean sociosexual behavior, but a consistent association with inferiority frustration. In this model, inferiority was positively linked to sociosexual behavior, β = .14, *SE* = .05, Bayesian 95% CI = [.06, .21], 2log(BF) = 13.81.

Second, when the associations with sociosexual attitudes were examined, the random-intercept models were found to be the best, indicating cross-cultural differences in mean sociosexual attitudes, but similar associations with both superiority enjoyment and inferiority frustration. Superiority, β = .17, *SE* = .04, Bayesian 95% CI = [.10, .24], 2log(BF) = Infinite, and inferiority were both positively related with sociosexual attitudes, β = .09, *SE* = .04, Bayesian 95% CI = [.02, .16], 2log(BF) = 8.32.

For sociosexual desire, the best models were the random-intercept models (M2) for both ICS subscales, indicating cross-cultural differences in mean levels of sociosexual desire, but consistent positive associations with both ICS components. Both superiority enjoyment, β = .25, *SE* = .04, Bayesian 95% CI = [.18, .32], 2log(BF) = Infinite, and inferiority frustration were positively related to sociosexual desire, β = .19, *SE* = .04, Bayesian 95% CI = [.11, .26], 2log(BF) = Infinite.

#### Cross-Country Comparison in Intrasexual Competition (H7)

**Fig. 1:**
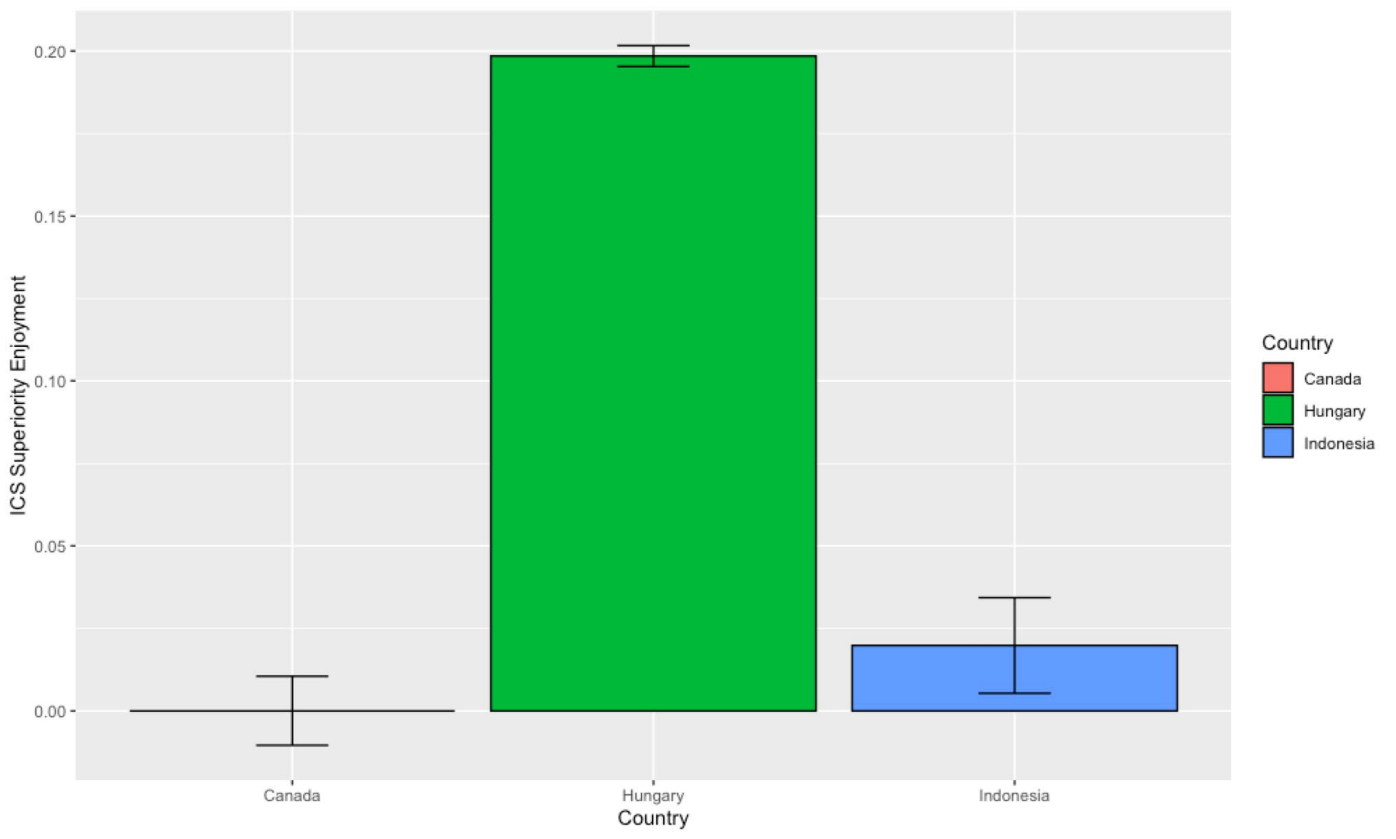
Comparison of Intrasexual Competition Scale superiority between countries. *Note*: *: *p* < .05. **: *p* < .01. All *p*-values were FDR corrected (Color figure online)

**Fig. 2:**
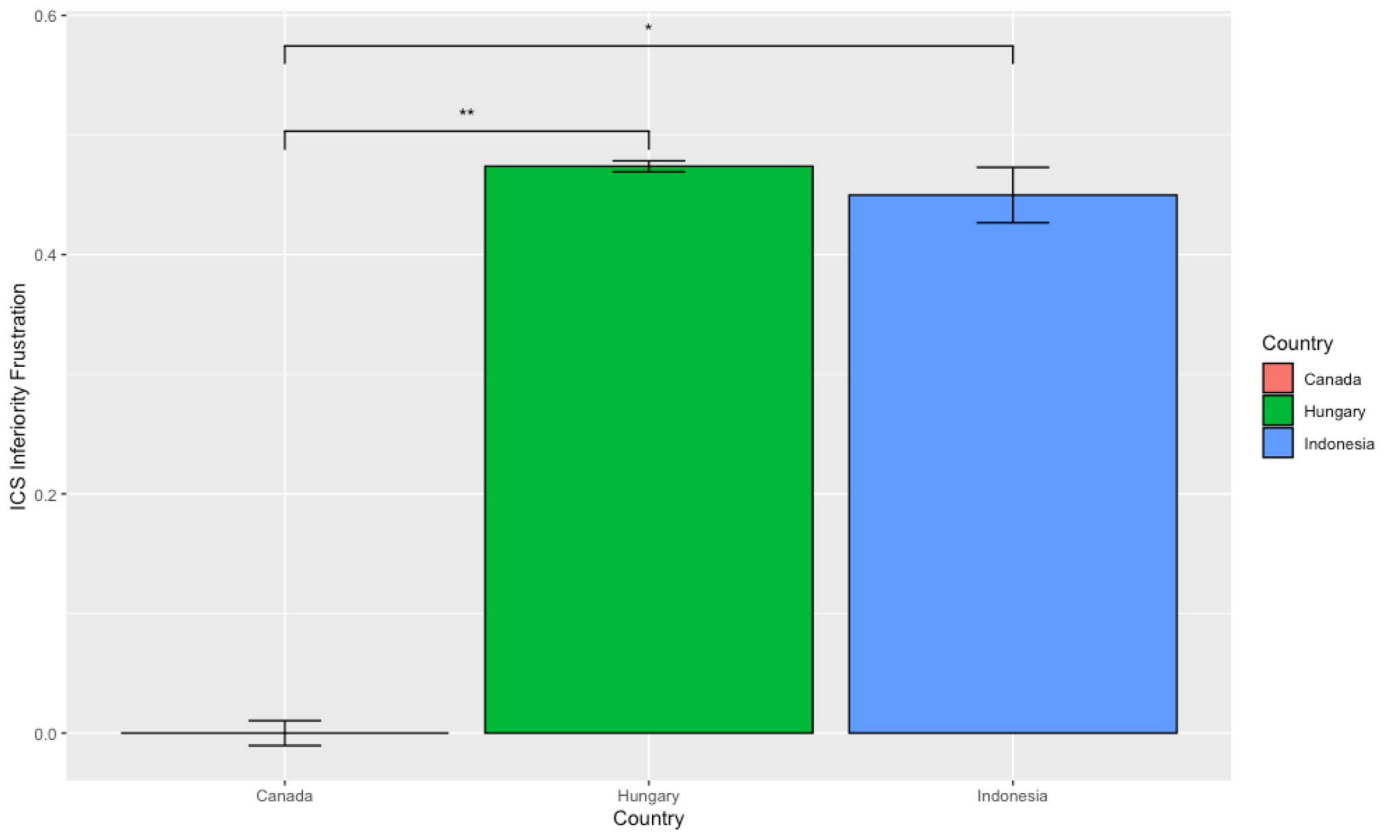
Comparison of Intrasexual Competition Scale inferiority between countries. *Note*: *: *p* < .05. **: *p* < .01. All *p*-values were FDR corrected (Color figure online)

To test H7, we compared the latent group means between Canada, Hungary, and Indonesia via measurement alignment. When we compared the aligned latent group means in ICS superiority after FDR correction, no significant international differences were found (see Fig. 1). In contrast, we found several pairwise differences in the latent group mean scores for ICS inferiority after correction (see Fig. 2). Canada demonstrated the significantly lower mean inferiority frustration compared with Hungary and Indonesia. However, the difference between Hungary and Indonesia was non-significant. These findings indicate that cultural differences emerged only for inferiority frustration, whereas superiority enjoyment showed no cross-national variation.

## Discussion

The current study expands our understanding of the psychology behind same-sex competition and provides a needed comparative analysis of intrasexual competition across cultures. Despite numerous empirical investigations on intrasexual competition using geographically and culturally diverse samples, this study is the first to provide comparative data for three countries regarding the ICS (Buunk & Fisher, 2009). In line with recent research concerning the ICS, we examined a two-factor model for intrasexual competitiveness: superiority enjoyment and inferiority frustration (Albert et al. 2022). Additionally, we examined and validated the ICS in both Hungarian and English through measurement invariance testing and alignment. The study revealed several novel results which are discussed below.

Taken together, the findings suggest that although the average levels of several examined constructs differed between countries, the associations between intrasexual competition and related psychological characteristics were largely similar across the cultural contexts studied. In other words, individuals who reported higher levels of intrasexual competition tended to show comparable patterns of sensation seeking, aggression, appearance enhancement, sexual motivation, and sociosexuality regardless of country. This pattern points to a degree of cross-cultural robustness in the psychological correlates of intrasexual competition, despite variation in baseline levels across societies.

### Intrasexual Competition and Sensation Seeking

A positive correlation was found between sensation seeking and the superiority enjoyment aspect of intrasexual competition, which supported our hypothesis. Our prediction was based on the findings of Buunk and Fisher (2009), as well as Buunk et al. (2017), who discovered a connection between intrasexual competition and the personality trait of extraversion, a construct that shows substantial conceptual overlap with sensation seeking. Although extraversion is not synonymous with sensation seeking, research suggests a close relationship between the two (e.g., Aluja et al. 2003; Johnson, 2021; Zuckerman et al. 1978), which is more characteristic of men than women.

### Intrasexual Competition and Aggression

Overall, support was found for H2: Intrasexual competition was positively linked with a tendency toward aggressiveness. Positive associations were supported between superiority enjoyment and physical and verbal aggression. However, no correlation between anger and superiority enjoyment was found. This finding contradicted our original hypothesis and suggests that the emotion of anger does not reliably covary with this aspect of intrasexual competitiveness. A positive correlation between hostility and superiority enjoyment was also supported.

The results regarding aggression mirror what was observed earlier with sensation seeking: Higher intrasexual competition was associated with higher levels of aggression. These findings may be explained by the possibility that individuals high in intrasexual competition are more likely to engage in aggressive responses when navigating competitive interpersonal contexts.

### Intrasexual Competition, Beauty-Enhancing Behavior, and Openness to Cosmetic Surgery

In support of H3, we found a positive association between the frequency of beauty-enhancing behavior and intrasexual competition, specifically the superiority enjoyment component. This finding supports the notion that engaging in appearance-enhancing behaviors aimed at increasing perceived attractiveness can function as a strategy through which individuals navigate intrasexual rivalry and competitive social contexts (Blake et al. 2020).

A substantial body of research has emphasized the role of physical appearance as a salient domain of intrasexual competition, particularly in situations where attractiveness serves as a meaningful cue for mate value and social comparison (Cashdan, 1998; Fink et al. 2014; Vaillancourt & Sharma, 2011). More broadly, environmental and socioeconomic conditions characterized by heightened competition and inequality have been shown to intensify appearance-related investments and self-presentation strategies (Blake et al. 2018; Blake & Brooks, 2019). Within such contexts, individuals high in intrasexual competitiveness may be especially sensitive to cues signaling rival attractiveness, which can activate appearance-focused competitive responses. Accordingly, beauty-enhancing behaviors can be understood as one of several strategies through which individuals seek to improve their competitive standing when facing perceived same-sex rivals (Blake et al. 2020; Davis & Arnocky, 2022; Wang et al. 2021). Supporting this interpretation, prior research has demonstrated positive associations between intrasexual competition and appearance-related social comparison, including comparisons occurring in online and social media environments (Hendrickse et al. 2017).

In support of H4, we found that superiority enjoyment was positively associated with all three aspects of openness to cosmetic surgery (Interpersonal, Social, and Consider). These findings are consistent with earlier work reporting associations between intrasexual competitiveness and openness to cosmetic procedures across diverse samples (Arnocky & Piché, 2014). Rather than reflecting sex-specific mechanisms, these associations may indicate broader processes whereby individuals who experience competitive advantage or enjoyment in rivalrous contexts are more willing to consider appearance-modifying strategies that could enhance perceived attractiveness, social status, or mate value. Physical appearance can function as a multifaceted signal conveying information related to health, vitality, and social standing, which may be relevant across a variety of social and mating contexts (Hanson Frieze, 2006; Soler et al. 2014). From this perspective, openness to cosmetic surgery may represent a flexible, appearance-based strategy that individuals high in intrasexual competitiveness are more inclined to adopt when such modifications are perceived as advantageous in competitive environments.

### Intrasexual Competition and Sexual Motivation

As predicted (H5.1), a positive correlation was found between more self-centered sexual motivation (personal goal attainment) and the superiority enjoyment component of intrasexual competition. Furthermore, in support of H5.2, a positive correlation was supported between more partner-centered sexual motivation (relational reasons) and both superiority enjoyment and inferiority frustration. Moreover, in line with our hypothesis (H5.3), positive associations between using sex as coping with emotional difficulties with both aspects of intrasexual competition were found.

These findings align with prior research suggesting that intrasexual competitiveness is associated with heightened sexual motivation and strategic engagement in sexual behavior (Buunk & Fisher, 2009). Heightened endorsement of specific sexual motives may facilitate more effective competitive strategies in short-term mating contexts, particularly when such strategies are perceived as advantageous. Prior research suggests that engaging in short-term sexual behavior can confer various benefits, including access to material or social resources (Gangestad & Simpson, 2000). From this perspective, sexual motivation may play an important role in shaping how individuals navigate competitive dynamics with same-sex rivals.

An alternative interpretation of the observed pattern is that individuals high in intrasexual competition may display a generally elevated openness toward sexual engagement, endorsing a wide range of reasons for having sex. Rather than undermining the meaningfulness of specific motivational dimensions, this pattern may reflect a more instrumental or flexible orientation toward sex, whereby sexual behavior is perceived as a viable means for achieving multiple interpersonal, emotional, or self-regulatory goals. From this perspective, the presence of positive associations across motivational subscales is theoretically consistent with an intrasexually competitive mindset, characterized by heightened readiness to deploy available strategies—sexual behavior among them—when navigating rivalry with same-sex peers.

### Intrasexual Competition and Sociosexuality

Positive correlations were supported between all three factors of sociosexuality (behavior, attitudes, and desire) with both superiority enjoyment and inferiority frustration. However, the pattern of associations varied across the three sociosexual dimensions, particularly in terms of model structure and effect magnitude. These results provide support for H6.

Davis et al. (2023a, 2023b) reported significant associations between intrasexual competition and unrestricted sociosexuality across samples, suggesting that sociosexual orientation may be meaningfully linked to competitive mating strategies. However, findings in this area have been mixed. For example, Buunk and Fisher (2009) observed a positive association in a Dutch student sample but not in a Canadian one, whereas other studies have reported null or context-dependent associations (Fiacco et al. 2019; Figueroa et al. 2020). Taken together, these inconsistencies highlight the need for further research to clarify the conditions under which sociosexuality and intrasexual competition are related.

### Cross-Country Differences in Intrasexual Competition

When conducting comparative analyses of the two subscales of intrasexual competition, we found no difference between Canada, Hungary, and Indonesia for superiority enjoyment. However, regarding inferiority frustration, Canadian respondents reported the lowest competition scores on average, whereas there was no difference between Hungarian and Indonesian scores. This result contradicts the hypothesis predicting that individualistic-oriented, loose-cultured Canadian participants would report higher intrasexual competition scores compared to collectivist-oriented tight-cultured Indonesian participants. Furthermore, with the Hungarian scores differing from the Canadian scores and not the Indonesian scores, the result contradicted H7.

A pivotal aspect distinguishing cultures is the continuum between individualism and collectivism (Hofstede, 1980, 1981). Societies exhibiting pronounced collectivism emphasize strong interpersonal bonds within their community, whereas societies leaning toward individualism prioritize self-reliance and self-governance (Hofstede, 2001). An alternative perspective suggests that cultures can be differentiated based on the extent of tightness or looseness, determined by the strength of societal norms and the measures governing and penalizing behaviors (Pelto, 1968; Witkin & Berry, 1975). In close-knit societies, norms are explicit and strict consequences are imposed on those diverging from them, emphasizing the importance of knowing and adhering to these norms within such cultures (Baldwin & Mussweiler, 2018; Gelfand et al. 2011; Uz, 2015).

Although it is challenging to precisely define Hungarian values compared to the cultural characteristics of Canada and Indonesia, previous research implies that Hungarian culture combines both Eastern and Western traits in economic (Falkné Bánó, 2002), family (Dupcsik & Tóth, 2015), sexuality (Meskó et al. 2024), and broader societal values (Keller, 2010). The results of the current study paint a picture that in terms of intrasexual competition, Hungary bears more similarity to Indonesia than to Canada.

Our findings are intriguing considering previous Indonesian studies on sexuality. Hartoyo and Abraham (2015) reported more conservative attitudes toward cybersex openness compared to Western norms. Khaerina and Abraham (2014) highlighted that authoritarian social/political norms may have a limiting impact on the sexual development of adolescents. Abraham and Rahardjo (2015) also reported that Indonesian urban adolescents characterized more by psychopathic personality traits, like their Western counterparts, were more open to premarital sex than those less characterized by this personality trait. These studies illustrate that cultural norms regarding sexuality in different countries can lead to varied expressions of intensity and pathways in sexual behaviors.

An additional pattern emerging across analyses was that superiority enjoyment tended to show stronger and more consistent associations with the examined psychological constructs than inferiority frustration. This asymmetry may reflect meaningful differences in the motivational and functional nature of the two components of intrasexual competition. Superiority enjoyment captures an approach-oriented, positively reinforcing engagement in competition, consistent with broader distinctions between approach- and avoidance-based motivational systems (e.g., Elliot & Thrash, 2002; Gray, 1987), which may be more directly linked to active behavioral and self-regulatory strategies such as sensation seeking, aggression, appearance enhancement, and unrestricted sexual motivation. In contrast, inferiority frustration reflects a more reactive, affectively negative response to perceived rivalry, which may be less consistently translated into outward behavioral strategies. From this perspective, the stronger associations observed for superiority enjoyment are theoretically consistent with its role as an active driver of competitive behavior rather than merely an emotional reaction to threat.

### Limitations and Future Directions

The current study addressed the need for more cross-cultural work on intrasexual competition and related facets of human mating psychology. However, due to the exploratory nature of the study, the results cannot provide a complete picture of the mechanisms through which examined factors influence competition for mating partners among individuals of the same sex.

The cross-sectional nature of the current research is also a notable limitation. Self-report questionnaires may introduce bias, and future studies should clarify the reproducibility of measurements using the ICS and different factor structures for the scale. Recently, neither Albert et al. (2022) nor Jonason and colleagues (2023) were able to replicate the original ICS (Buunk & Fisher, 2009) single-factor solution, and instead found the two- and three-factor solutions to be more appropriate. More research, like this current work, is needed to further examine the reliability and validity of these newer models for intrasexual competition.

We also need to consider the disproportional sample sizes across different countries. Only 96 participants were recruited from Indonesia, whereas 124 and 272 participants were recruited from Canada and Hungary, respectively. The small sample size in Indonesia might have contributed to the larger standard error in this group, and perhaps the non-significant correlations in the analysis with the inferiority frustration component of intrasexual competitiveness. Therefore, further investigations with more representative sampling are necessary for a more precise portrayal of the relationships explored in this study. It would also be prudent to examine cultural-level variables that might help to account for differences between nations, such as the level of income inequality (Blake et al. 2018).

Although sex differences are theoretically important in evolutionary accounts of mating-related processes, the present study did not conduct sex-stratified analyses. This decision was primarily motivated by the strongly unbalanced sex distribution of the sample (approximately 75% women, 20% men, with a small proportion of non-binary participants), which would have resulted in highly unequal and, in some cases, insufficient group sizes. Such imbalance substantially reduces statistical power and can yield unstable parameter estimates, inflated standard errors, and potentially misleading conclusions when group comparisons are conducted (Gelman & Hill, 2007; Maxwell et al. 2018; Tabachnick & Fidell, 2019).

In earlier exploratory analyses conducted during manuscript preparation, sex-specific models did not produce reliable or interpretable effects, likely due to these sample characteristics. Given these considerations, we opted to focus on intrasexual competition as an individual-difference construct operating within individuals rather than between sexes. This approach is consistent with prior work using the Intrasexual Competition Scale, which has frequently examined individual variability without sex-stratified modeling (e.g., Albert et al. 2022; Buunk & Fisher, 2009). Future research using more balanced samples will be well positioned to test whether and how the observed associations differ as a function of sex.

Given the absence of preregistration, findings—particularly those that are novel or unexpected—should be interpreted with appropriate caution.

### Conclusion

The current study is unique in measuring the associations of intrasexual competition with psychological constructs with an international sample. These constructs include sensation seeking, aggression, beauty-enhancing behaviors, openness to cosmetic surgery, sexual motivation, and sociosexuality. The study takes an important step toward understanding the phenomenon of intrasexual competition across different cultures or demographic groups, alongside individual-level factors. The study's primary strength lies in its cross-cultural nature and the use of multiple questionnaires, allowing for the systematic examination of impacts of individual and cultural variables. This allows for a more nuanced understanding of intrasexual competition, providing key insights into the psychology of human mate selection. The findings may also result in more effective methods to counterbalance potential negative psychological consequences associated with sexual competition.

The present research contributes to the literature on intrasexual competition by replicating some previous results wherein the same measurement instrument was used (the ISC; Buunk & Fisher, 2009), including the positive links between intrasexual competition and acceptance of cosmetic surgery and sociosexuality. Novel relationships using the ISC were also supported, such as the positive relation between intrasexual competition and sensation seeking. We further established relations not previously investigated between intrasexual competition and beauty-enhancing behavior and sexual motivation. A cross-cultural analysis of the ISC across three different nations (Canada, Hungary, and Indonesia) was also provided. These results were also supported by newer and more precise statistical methods (Bayesian approach) used to analyze the data.

## Supplementary Information

Below is the link to the electronic supplementary material.

Supplementary file1 (DOCX 17 KB)

## Data Availability

All survey materials, collected data, and R codes for analysis can be found on the Open Science Framework project website: 10.17605/OSF.IO/7A6QR.
