# Supplementary material for: A Cross-Cultural Examination of Intrasexual Competition: The Links Between Mating Competition, Aggression, Appearance Enhancement, and Sexuality
Source: Arch Sex Behav. 2026 Jun 22;55(5):2049–70. doi: 10.1007/s10508-026-03464-8 (PMC13427859; doi:10.1007/s10508-026-03464-8)
Supplement: Supplementary file 1 — Supplementary file1 (DOCX 17 KB) [file 10508_2026_3464_MOESM1_ESM.docx]

**Table S1**

*Means and Standard Deviations of the Study Variables (Overall and by Sex)*

| **Variable** | **Overall** | **Women** | **Men** |
| --- | --- | --- | --- |
| BSSS Sensation Seeking | 23.48 (6.73) | 23.02 (6.58) | 25.25 (7.04) |
| BPAQ Physical Aggression | 5.51 (2.56) | 5.34 (2.52) | 6.17 (2.61) |
| BPAQ Verbal Aggression | 6.66 (2.58) | 6.65 (2.58) | 6.64 (2.59) |
| BPAQ Anger | 7.75 (2.96) | 7.77 (3.00) | 7.69 (2.81) |
| BPAQ Hostility | 6.96 (2.86) | 6.99 (2.88) | 6.81 (2.77) |
| ACSS Intrapersonal | 20.03 (8.24) | 20.11 (8.02) | 19.74 (9.05) |
| ACSS Social | 12.35 (7.74) | 12.14 (7.58) | 13.09 (8.28) |
| ACSS Consider | 17.81 (10.03) | 18.24 (10.14) | 10.09 (9.40) |
| BEBS Total | 460.61 (533.20) | 465.19 (541.20) | 435.31 (495.28) |
| ISC Superiority Enjoyment | 3.70 (1.55) | 3.64 (1.54) | 3.92 (1.58) |
| ISC Inferiority Frustration | 2.23 (.99) | 2.23 (1.00) | 2.17 (.93) |
| YSEX Personal Goal Attainment | 9.24 (3.63) | 8.98 (3.47) | 10.15 (4.01) |
| YSEX Relational Reasons | 12.18 (5.09) | 11.98 (10) | 12.90 (5.05) |
| YSEX Sex as Coping | 8.43 (3.59) | 8.28 (3.58) | 8.95 (3.58) |
| SOI Behavior | 6.69 (4.95) | 6.27 (4.49) | 8.16 (6.08) |
| SOI Attitude | 14.57 (7.81) | 13.76 (7.84) | 17.65 (6.91) |
| SOI Desire | 8.68 (5.70) | 7.52 (4.84) | 12.97 (6.43) |

**Note**. Values represent means and standard deviations (M, SD). Sex was coded based on sex assigned at birth. Non-binary respondents were excluded from sex-specific descriptive statistics due to small group size.

BSSS = Brief Sensation Seeking Scale; BPAQ = Buss–Perry Aggression Questionnaire;

ACSS = Acceptance of Cosmetic Surgery Scale; BEBS = Beauty Enhancement Behavior Scale; ISC = Intrasexual Competition Scale; YSEX?-15 = 15-item version of Reasons for Having Sex Questionnaire; SOI-R = Revised Sociosexual Orientation Inventory.
